# For a Mad Dog’s Bite

**Published:** 1881-03

**Authors:** 


FOR A MAD DOG'S BITE.—*An Alleged Sure Cure that has Been Guarded as a Secret for Many Years.*—"Penny's Receipt for the Cure of Hydrophobia.—Take two upper and one under oyster shell well freshened and burnt to a lime, Roach allum, well burnt Bolarmenia or Dragons Blood pulverized, taken in middling good white wine, beer or ale, kept in a glass jar air tight. The Alicompan root must be dried and made fine, the shell lime and alicompain must be ⅛ the most, the other two articles half and half, all well mixed together and taken for man or beast weighing 160 pounds, one common tablespoonful or so in proportion to any heft; mix early in the morning, after being bit, in white wine, beer or ale, fasting four hours and neither eat nor drink and put some of the medicine on the wound; mix in some grease and bind it on tight and so on for two mornings running and the fourth morning take the third dose fasting each time as the above, and ardent spirits must not be drank for three months after."

JAMES PENNY'S RECEIPT:

"Frantzdale, Ulster County, April 28, 1837. To be more explicit the four articles are to be mixed in equal parts by measure except the lime and alicompain to be one-eighth the most each." This receipt is copied verbatim for what it may be worth.

